# Left Ventricular Mass Index and Pulmonary Artery Pressure in Patients with the Obstructive Sleep Apnea Syndrome

**Published:** 2016-01-13

**Authors:** Seyed Hashem Sezavar, Shokoufeh Hajsadeghi, Maral Hejrati, Mir Farhad Ghaleh Bandi

**Affiliations:** *Rasoul-e-Akram Hospital, Iran University of Medical Sciences, Tehran, Iran.*

**Keywords:** *Sleep apnea, obstructive*, *Heart ventricles*, *Pulmonary artery*

## Abstract

**Background: **Sleep apnea is accompanied by some cardiovascular complications. It has even been hypothesized that sleep apnea, itself, can induce some of these complications. Given such controversies, we assessed the left ventricular mass index (LVMI) and systolic pulmonary artery pressure in patients with sleep apnea.

**Methods:** Through convenience sampling, 56 patients with the obstructive sleep apnea syndrome (OSAS) were included in the present descriptive cross-sectional study. Patients with any past history of hypertension and diabetes mellitus were excluded. The apnea severity was assessed via the polysomnography-derived apnea-hypopnea index (AHI). All the patients underwent transthoracic echocardiography. In this cross-sectional study - data regarding age, gender, smoking, systolic and diastolic blood pressures, polysomnographic parameters (AHI, severity of disease, mean heart rate, mean oxygen saturation [SaO_2_], lowest SaO_2_, and duration of SaO_2_ below 90% [d.SaO_2_ < 90%]), and echocardiographic parameters (systolic pulmonary artery pressure and LVMI) were accumulated and processed.

**Results: **Fifty-two men and 14 women at a mean age of 49.29 ± 11.79 years participated in this study. Systolic and was significantly high in the severe group compared with the mild group (128.21 ± 9.73 mmHg vs. 119.23 ± 12.5 mmHg; p value = 0.007). The LVMI was increased parallel to an increase in the severity of the OSAS, but that increase was not statistically significant (p value = 0.161). The d.SaO_2_ < 90% was positively correlated with the LVMI, and this relationship remained true after adjustment for the body mass index (r = 0.27; p value = 0.042).

**Conclusion**: Severe OSAS was accompanied by a higher blood pressure. The LVMI did not differ significantly between the patients with the OSAS and those who did not suffer from other risk factors of cardiac diseases.

## Introduction

Sleep-disordered breathing includes the obstructive sleep apnea syndrome (OSAS) and the upper airway resistance syndrome. Epidemiological studies based on the general population or the community-based cohort between 30 and 60 years of age estimate that 2 to 5% of the population is affected by the OSAS.^1^ The OSAS is known as the upper respiratory tract occlusion leading to the complete or partial cessation of the air flow. Hypoxia and ventilatory efforts are the consequences of this condition, and transient arousal occurs to improve the air flow. An obstructive apnea is the absence of the air flow accompanied by ventilatory efforts for at least 10 seconds. A hypopnea is considered clinically significant if it is associated with a central nervous system arousal or with an oxyhemoglobin desaturation event (a decrease in blood oxygen saturation [SaO_2_] by 3 to 4% or more).^1^ The apnea-hypopnea index (AHI) is the total number of apneas and hypopneas per hour of sleep. The AHI is a common and useful index for evaluating the severity of the OSAS. Patients are classified into three groups based on their AHI: mild (AHI, 5 to 15), moderate (AHI, 15 to 30), and severe (AHI ≥ 30).^1^ Several causes have been mentioned to explain how the cardiovascular complications are related to the OSAS- including increased sympathetic activity in response to hypoxia and hypercapnia, which itself causes vasoconstriction, acute tachycardia, acute blood pressure elevation, and decrease in cardiovascular variability.^2^^, ^^3^ The OSAS is prevalent in patients with cardiovascular disease. It has been estimated that 50% of patients with hypertension, 33% with coronary artery disease, 50 to 60% with myocardial infarction, 30 to 40% with systolic heart failure, and 50% with atrial fibrillation requiring cardioversion suffer from the OSAS.^2^ On the other hand, some cardiovascular complications are presumed to be the consequences of the OSAS. This relationship may be caused by the OSAS comorbidities such as obesity and metabolic dysregulations. However, according to observational studies, the OSAS - itself - may lead to or worsen cardiovascular complications.

## Methods

Fifty-eight patients diagnosed with the OSAS via polysomnography were enrolled in this cross-sectional study, conducted from March 2011 to May 2013. Unfortunately, recruiting only a small population with sleep disorders having undergone polysomnography and accepted to undergo echocardiography was one of the major limitations to our study. The patients underwent polysomnography in Nour Sleep Disorders Clinic, followed by echocardiography in Rasoul-e-Akram Hospital, Tehran, Iran. The study was performed in accordance with the Declaration of Helsinki and subsequent revisions. Furthermore, the Ethics Committee of Iran University of Medical Sciences approved the protocol before the study was commenced. Two patients were excluded from the study because of the diagnosis of severe mitral stenosis and myopathy due to frequent premature ventricular contractions on echocardiography. Patients with hypertension, diabetes mellitus, chronic kidney disease, cardiomyopathy, and any past history of cardiopulmonary diseases as well as individuals with the AHI < 5 assumed as the normal population1 were excluded from the study. Transthoracic echocardiography was performed by a single fellow of echocardiography. The echocardiographer was blinded to the patients' polysomnography report. A manual check list was devised for data collection. The data encompassed age, gender, smoking, systolic and diastolic blood pressures, polysomnographic parameters (the AHI, severity of disease, mean heart rate, mean SaO_2_, lowest SaO_2_, and duration of SaO_2_ (d.SaO_2_) < 90%, and echocardiographic parameters (systolic pulmonary artery pressure and left ventricular mass index [LVMI]). The (LVMI) was calculated according to the Devereux formula. The LVMI was calculated by the correction of the left ventricular mass (LVM) for the body surface area. The normal threshold for the LVMI was defined < 96 for women and < 116 for men.^4^ The patients were divided into three groups according to their AHI: mild (AHI, 5 to 15), moderate (AHI, 15 to 30), and severe (AHI ≥ 30). For the statistical analyses, the statistical software SPSS version 13 for Windows (SPSS Inc., Chicago, IL) was used. The quantitative variables were presented as means ± standard deviations for the normally distributed variables and as medians (interquartile ranges) for the variables without normal distribution. The categorical data were presented as numbers and percentages. The categorical data were compared using the chi-square test, whereas the quantitative ones were compared using the one-way analysis of variance and the Kruskal–Wallis test, as appropriate. Relationships were assessed using the Pearson correlation test, Spearman test, or Kendall test based on their distribution. All p values were two-tailed, and a p value < 0.05 was considered statistically significant.

## Results

The 56 eligible patients recruited in this study were comprised of 42 (75%) men and 14 (25%) women. The mean age was 49.29±11.79 years old, and the mean body mass index (BMI) was 28.12 ± 3.59 kg/m^2^ (Table 1). Thirteen (23.2%) patients had mild OSAS with a mean AHI of 7.23 ± 1.61 (Group I). Fifteen (26.8%) patients were in the moderate group (Group II) (AHI, 23.05 ± 3.97) and 28 (50%) were in the severe group (Group III) (AHI, 61.17 ± 24.07). The demographic data and echocardiographic parameters were compared between the groups. 

**Table 1:** Study population’s characteristics*

Age (y)	49.25±11.79
BMI (kg/m^2^)	28.12±3.59
PAP(mmHg)	29.38±3.31
SBP (mmHg)	125.18±10.08
DBP (mmHg)	74.46±7.48
AHI (per hour)	29.5 (16.78-61.90)
Mean SaO_2_ (%)	92.85 (91.08-93.95)
Lowest SaO_2_ (%)	79.50 (76.00-86.00)
d.SaO_2_ 90% (min)	17.15 (2.00-60.20)
LVMI (g/m^2^)	77.41 (66.38-91.99)

BMI, Body mass index; PAP, Pulmonary artery pressure; SBP, Systolic blood pressure; DBP, Diastolic blood pressure; AHI, Apnea-hypopnea index; SaO_2_, Saturation of oxygen; d.SaO_2_ < 90%, Duration of oxygen saturation below 90%; LVMI, Left ventricular mass index

* Data are presented as mean±SD or median (interquartile ranges).

**Table 2:** Comparison of the variables between the groups^*^

	Mild Sleep Apnea	Moderate Sleep Apnea	Severe Sleep Apnea	P Value
Age (y)	74.31±10.78	47.93±13.8	50.93±11.25	0.581
BMI (kg/m^2^)	26.63±3.36	27.32±2.01	29.25±4.06	0.007
PAP (mm Hg)	29.62±3.20	29.33±3.71	29.29±3.25	0.953
SBP (mm Hg)	119.23±12.05	124.67±6.39	128.21±9.73	0.037
DBP (mm Hg)	70.00±9.12	76.67±7.23	75.36±6.07	0.487
LVMI	71.8 (63.35-86.25)	74.6 (63.84-86.02)	80.53 (71.46-105.23)	0.095

BMI, Body mass index; PAP, Pulmonary artery pressure; SBP, Systolic blood pressure; DBP, Diastolic blood pressure; LVMI, Left ventricular mass index

* Data are presented as mean±SD or median (interquartile ranges).

No significant differences were detectable between the groups concerning age (p value = 0.581), whereas systolic blood pressure was significantly high in the severe group compared with the mild group (128.21 ± 9.73 mmHg vs. 119.23 ± 12.5 mmHg; p value = 0.007). Additionally, a lower diastolic blood pressure was demonstrated in the mild group than in both the moderate and severe groups (p value = 0.487) (Table 2). The BMI differed significantly between Group I (26.63 ± 3.36 kg/m^2^) and Group III (29.25 ± 4.06 kg/m^2^), (p value = 0.022). A partial correlation was used with controlling for the body mass index (BMI). There were no significant differences between the groups regarding systolic pulmonary artery pressure (p value = 0.953). The LVMI was increased parallel to an increase in the OSAS severity, but that increase failed to constitute statistical significance (p value = 0.095) (Table 2). Six patients were diagnosed with LV hypertrophy; 4 (66.6%) of these patients were in Group III. Significant correlations were detected between the BMI and each of the hypoxic parameters - including the AHI, mean SaO_2_, lowest SaO_2_, and d.SaO_2_ < 90% (p value = 0.002, p value = 0.010, p value = 0.001, and p value < 0.001, respectively). Similarly, a significant positive correlation was detected between the BMI and systolic blood pressure (p value = 0.003). Although the initial analysis showed a negative correlation between systolic blood pressure and mean SaO_2_ (r = - 0.29; p value = 0.020), a negative correlation between systolic blood pressure and lowest SaO_2_ (r = - 0.27; p value = 0.041), and a positive correlation between systolic blood pressure and d.SaO_2_ < 90% (r = 0.34; p value = 0.009), these relationships were not confirmed after adjustment for the BMI. The LVMI was positively correlated with d.SaO_2_ < 90%, even after adjustment for the BMI (r = 0.27; p value = 0.042). There were no statistically significant correlations between the LVMI and the other hypoxemic parameters - including the AHI (r = 0.32), mean SaO_2_ (r = -0.24), and lowest SaO_2_ (r = -0.19).

## Discussion

Fifty-six patients with the OSAS participated in our cross-sectional study and underwent transthoracic echocardiography. The initial analysis showed correlations between systolic blood pressure and hypoxemic parameters, but these relationships were not confirmed after adjustment for the BMI in our study. A 4-year study on 709 subjects demonstrated that the OSAS was a risk factor for elevating blood pressure after adjustment for age, gender, neck circumference, and the BMI.^5^ Another 4-year study established a rise in blood pressure and its strong and independent relationship with the OSAS.^6^ Based on our data, the BMI differed significantly in between Group I (26.63 ± 3.36 kg/m^2^) and Group III (29.25 ± 4.06 kg/m^2^), and there were significant correlations between the BMI and each of the hypoxemic parameters - including the AHI, mean SaO_2_, lowest SaO_2_, and d.SaO_2_ < 90%.^7^ Our results also revealed no significant differences between the groups apropos pulmonary artery pressure. The LVMI was increased in tandem with a rise in the OSAS severity in our study; the increase, however, was not statistically significant. The LVMI was positively correlated with the d.SaO_2_ < 90%, even after adjustment for the BMI. This relationship was not true between the LVMI and the other hypoxemic parameters - including the AHI, mean SaO_2_, and lowest SaO_2_. In our study, 6 patients had LV hypertrophy; 4 (66.6%) of these patients were in the severe apnea group. It has been previously reported that the OSAS is an independent predictor for the occurrence of LV hypertrophy.^8^ Nonetheless, a study performed between 1990 and 1998 on patients with the OSAS demonstrated that the LV hypertrophy in these patients was associated with higher age, obesity, and hypertension.^9^ Dursunoglu et al^10^ reported that the LVMI in patients with the AHI ≥ 15 was higher than that in individuals with the AHI < 15. Another study on 28 patients with the OSAS without any past history of cardiovascular diseases showed LV hypertrophy in 21%; nevertheless, the effect of age and the BMI were not taken into consideration in this study (Gorzewska A, Drozdowski J, Jerzemowska A, Cynowska B, Porzezinska M, Kedziora K, Grzenkowicz MS, Slominski JM, Cardiovascular complications in obstructive sleep apnoea: Early signs of cardiovascular system dysfunction in obstructive sleep apnea (OSA) patients before clinical manifestation, Hall 2009,15:3686). It has been believed that LV hypertrophy in patients with the OSAS was independent of the other risk factors and that there was a relationship between this complication and O_2_ desaturation (Angeles Fernandez Jorge M, Luis Delgado J, Ruiz, Gloria Sobrino T, Antonio Iglasias J, Cardiovascular complications in obstructive sleep apnoea: Leftventricular hypertrophy (LVH) and diastolic dysfunction (DD) in obstructive sleep apnea (SAHS) patients. Hall 2009, 15:3698.). In another study on 50 patients with the OSAS, all the patients who were diagnosed with LV hypertrophy suffered from hypertension and the LVMI was strongly associated with the d.SaO_2_ < 90%.^11^ According to another study, the relationship between the severity of the OSAS and LV thickness was weakened by an increase in age and smoking and was invigorated by hypertension.^12^

## Conclusion

Severe OSAS is accompanied by a higher blood pressure. Whereas the LVMI did not differ significantly between the patients with the OSAS who were not heavy smokers and did not suffer from hypertension and/or diabetes mellitus, LV hypertrophy occurred more frequently in those with severe OSAS (66%) than in those who belonged to the mild and moderate groups. Finally, an increase in the LVMI was correlated with a rise in the d.SaO_2_ < 90%.

## Notes:

***This paper should be cited as:*** Sezavar SH, Hajsadeghi S, Hejrati M, Ghaleh Bandi MF. Comparison Study of the Left Ventricular Mass Index and Pulmonary Artery Pressure in Patients with the Obstructive Sleep Apnea Syndrome. J Teh Univ Heart Ctr 2016;11(1):11-14.
